# Alterations of 63 hub genes during lingual carcinogenesis in C57BL/6J mice

**DOI:** 10.1038/s41598-018-31103-3

**Published:** 2018-08-22

**Authors:** Hua Liu, Jianjiao Li, Ying Yang, Liu Liu, Lifu Yu, Minsong Tu, Ruihong Yuan, Wanyuan Yue, Qi Luo, Yonghua Ruan, Xiaoming Dai

**Affiliations:** 1 Department of Oral and Maxillofacial surgery, the Fourth Affiliated Hospital of Kunming Medical University, No. 176, Qinnian Road, Kunming city, Yunnan Province, China; 2 Maxillofacial Service of the Department of Plastic Surgery, the First Affiliated Hospital of Kunming Medical University, No. 295, Xichang Road, Kunming city, Yunnan Province, China; 3 Department of Pathology, the First Affiliated Hospital of Kunming Medical University, No. 295, Xichang Road, Kunming city, Yunnan Province, China; 4 Department of Pathology, Kunming Medical University, No. 1168, Chunrongxi Road, Chenggong District, Kunming city, Yunnan Province, China

## Abstract

To identify potential biomarkers of lingual cancer, 75 female C57BL/6J mice were subjected to 16-week oral delivery of 4-nitroquinoline-1-oxide (4NQO; 50 mg/L), with 10 mice used as controls. Lingual mucosa samples representative of normal tissue (week 0) and early (week 12) and advanced (week 28) tumorigenesis were harvested for microarray and methylated DNA immunoprecipitation sequencing (MeDIP-Seq). Combined analysis with Short Time-series Expression Miner (STEM), the Cytoscape plugin cytoHubba, and screening of differentially expressed genes enabled identification of 63 hub genes predominantly altered in the early stage rather than the advanced stage. Validation of microarray results was carried out using qRT-PCR. Of 63 human orthologous genes, 35 correlated with human oral squamous cell carcinoma. KEGG analysis showed “pathways in cancer”, involving 13 hub genes, as the leading KEGG term. Significant alterations in promoter methylation were confirmed at Tbp, Smad1, Smad4, Pdpk1, Camk2, Atxn3, and Cdh2. HDAC2, TBP, and EP300 scored ≥10 on Maximal Clique Centrality (MCC) in STEM profile 11 and were overexpressed in human tongue cancer samples. However, expression did not correlate with smoking status, tumor differentiation, or overall survival. These results highlight potentially useful candidate biomarkers for lingual cancer prevention, diagnosis, and treatment.

## Introduction

Lip and oral cancers represent the fifteenth most common malignant tumors worldwide, with 410,304 new cases reported in 2015^1^. Tongue squamous cell carcinomas (SCCs) account for approximately 30−40% of all cases of malignancies of the oral cavity and pharynx^2^. The 5-year survival rate for oral SCCs (HOSCCs) remains low. Therefore, development of new prophylactic, diagnostic, and therapeutic strategies to reduce the morbidity and mortality associated with this type of tumor is essential^3^.

Pathologically, HOSCCs usually progress through oral premalignant lesions (OPL), and develop stepwise from hyperplasia to dysplasia, and eventually to invasive SCCs^4^. Given the described stepwise progression of oral carcinogenesis, gene expression patterns and aberrant DNA methylation at each stage should be examined to identify clinically useful biomarkers. Nonetheless, few studies have addressed the dynamics of genetic changes in oral cancer^5,6^.

Recently, the development of bioinformatics tools has paralleled the explosive increase in available clinical and experimental data. Algorithms have been designed to schematize nodes (molecular entities such as genes, proteins, metabolites, or gene transcripts) interconnected by edges that reflect the functionality of biological systems and processes. The interrelation between connectivity and indispensability of a given node signifies its importance, which is largely assessed by its topological centrality in a biological network. Highly connected nodes are termed “hubs,” which maintain the structure of protein–protein interaction networks (PIN). According to the centrality–lethality rule, the whole PIN will collapse if hubs are removed^7,8^.

Murine lingual SCC induced 4NQO is an ideal model of human tongue cancer, as it reproduces the sequential histopathological lesions that occur in patients^9^.

Therefore, we induced lingual SCCs in C57BL/6 J mice using 4NQO and assessed gene expression changes throughout tumor induction and progression. Further, we assayed protein expression in human tongue SCC (HTSCC) specimens. The present data provide new insights into stage-specific gene expression alterations during oral tumorigenesis and suggest potential biomarkers for early diagnosis and therapy.

## Results

### Experimental model of lingual SCC

**Figure 1:**
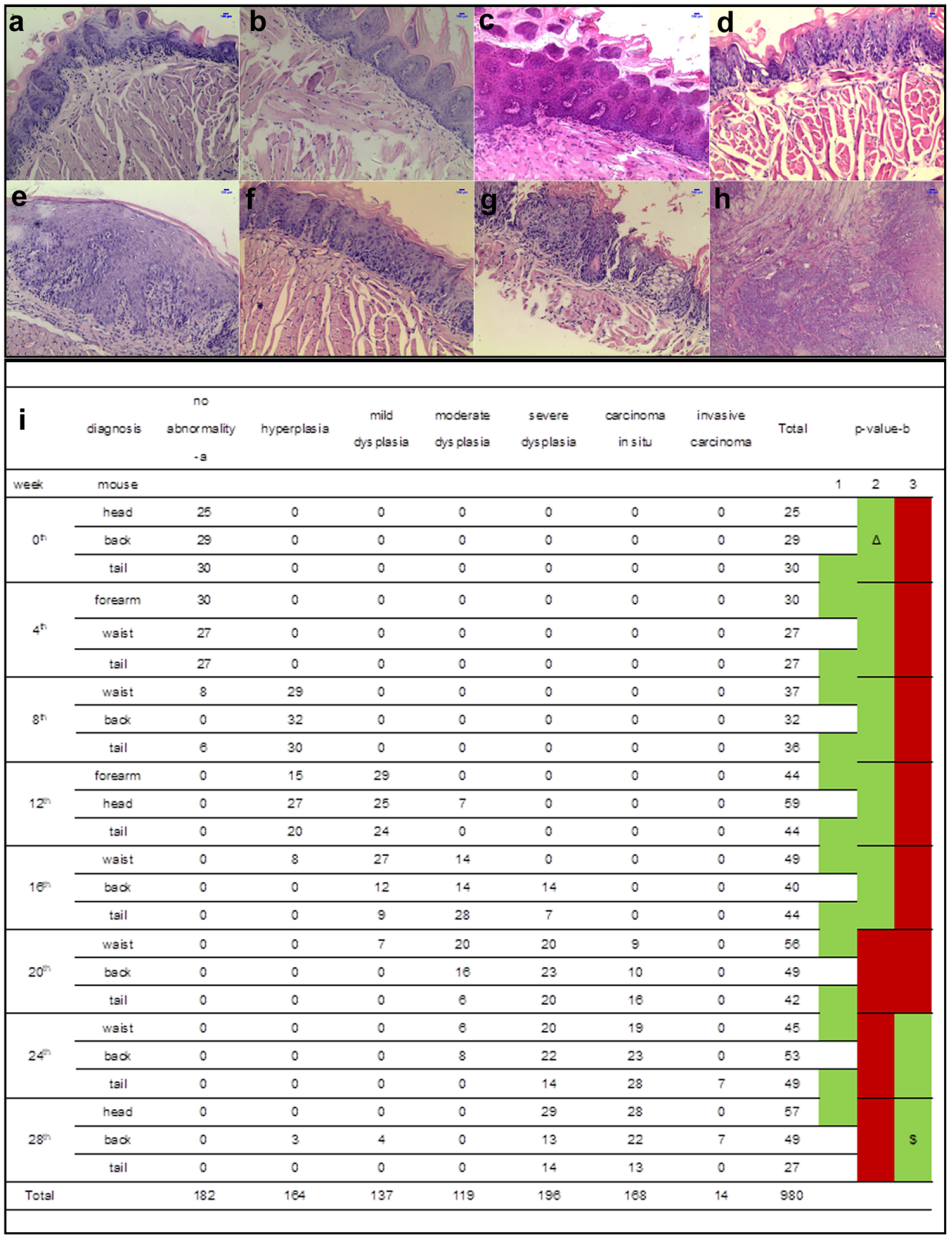
Pathological results of mouse model. The typical pathological results of lingual carcinogenesis in C57BL/6 J female mice induced by 4-NQO are manifested with a magnification of ×100 (**a–h**). The length of blue bar in the top right corner is 100 μm. (**a**) Sample from distilled water control group harvested at the 28th week. (**b**) Sample from propylene glycol control group harvested at the 28th week. (**c–h**) Samples from testing group. (**c**) Hyperplasia. (**d**) Mild dysplasia. (**e**) Moderate dysplasia. (**f**) Severe dysplasia. (**g**) Carcinoma *in situ*. (**h**) Invasive cancer. The pathological results present statistical significance in general (i, rank sum test, Kruskal-Wallis method, α = 0.05, H_C_ = 685, P < 0.005). In comparison with mice of the 28^th^ week, statistical differences were confirmed as early as in mice of the 20^th^ week (rank sum test, Nemenyi method, α = 0.05, P < 0.01). -a: each section with a result of NA was counted as one. (-**b:**) Results of pair-wise comparisons between groups. Green areas represented no statistical significance between groups (p > 0.05), while red ones demonstrated existence of statistical significance between groups (p < 0.05) in comparison with Δ (results of week 0) or $ (results of week 28). Column 1 demonstrated that there existed no statistical significance between any two adjacent groups. Column 2 and 3 manifested the groups with statistical differences compared with week 0 and week 28 individually.

Lingual SCC was induced in C57BL/6 J mice by 4NQO administration in drinking water for 16 weeks. Out of 85 mice, 84 were evaluated as one mouse in the SCC group died. Various kinds of lesions were identified (Fig. 1). A lesion was defined as pathologically abnormal epithelial area without interruptions by normal epithelium. For statistical analyses, hyperplasia and mild and moderate dysplasia were classified as lesions of early stage, whereas severe dysplasia, carcinoma *in situ*, and infiltrating carcinoma were grouped as advanced stage. The overall histopathological findings at different time points among SCC mice were significantly different. Pair-wise comparisons between groups indicated that comparison with 28^th^ week samples, statistical differences were confirmed as early as in the 20^th^ week (Fig. 1). Given that severe dysplasia and carcinoma *in situ* manifested as early as in the 16^th^ and 20^th^ weeks, respectively, it was not ideal to use samples of these two groups to represent early-stage carcinogenesis. Thus, 12-week post-SCC induction samples were chosen as “early stage” for further research.

### Gene expression profiling by microarray analysis

Samples from the 0^th^ week group (C), the 12^th^ week group (M), and the 28^th^ week group (E) were used for genome-scale microarray analysis. Raw data were submitted to Gene Expression Omnibus (GEO: GSE101469). Next, differentially expressed genes (DEGs) were screened. For M vs. C, 1 193 and 1 146 were up- and downregulated, respectively. A total of 1 125 out of 2 539 genes were upregulated between E and M. Comparison between E and C identified upregulation of 1 642 genes from a total of 2 482 DEGs (see Supplementary Table S1). GO analysis identified the top 10 GO terms with the highest enrichment score (ES) on the three domains (see Supplementary Fig. S1). The top 10 significantly enriched pathways are shown in Supplementary Fig. S2.

A total of 7 090 hub genes were classified into networks in the present experiment. The scores of hub genes by MCC varied from 0 to 367. The networks of the top 10 hub genes are illustrated in Supplementary Fig. S3. Of 7 090 hub genes with an MCC score ≥10, 987 (14%) were considered central elements in the biological networks and selected for further combination assays.

**Figure 2:**
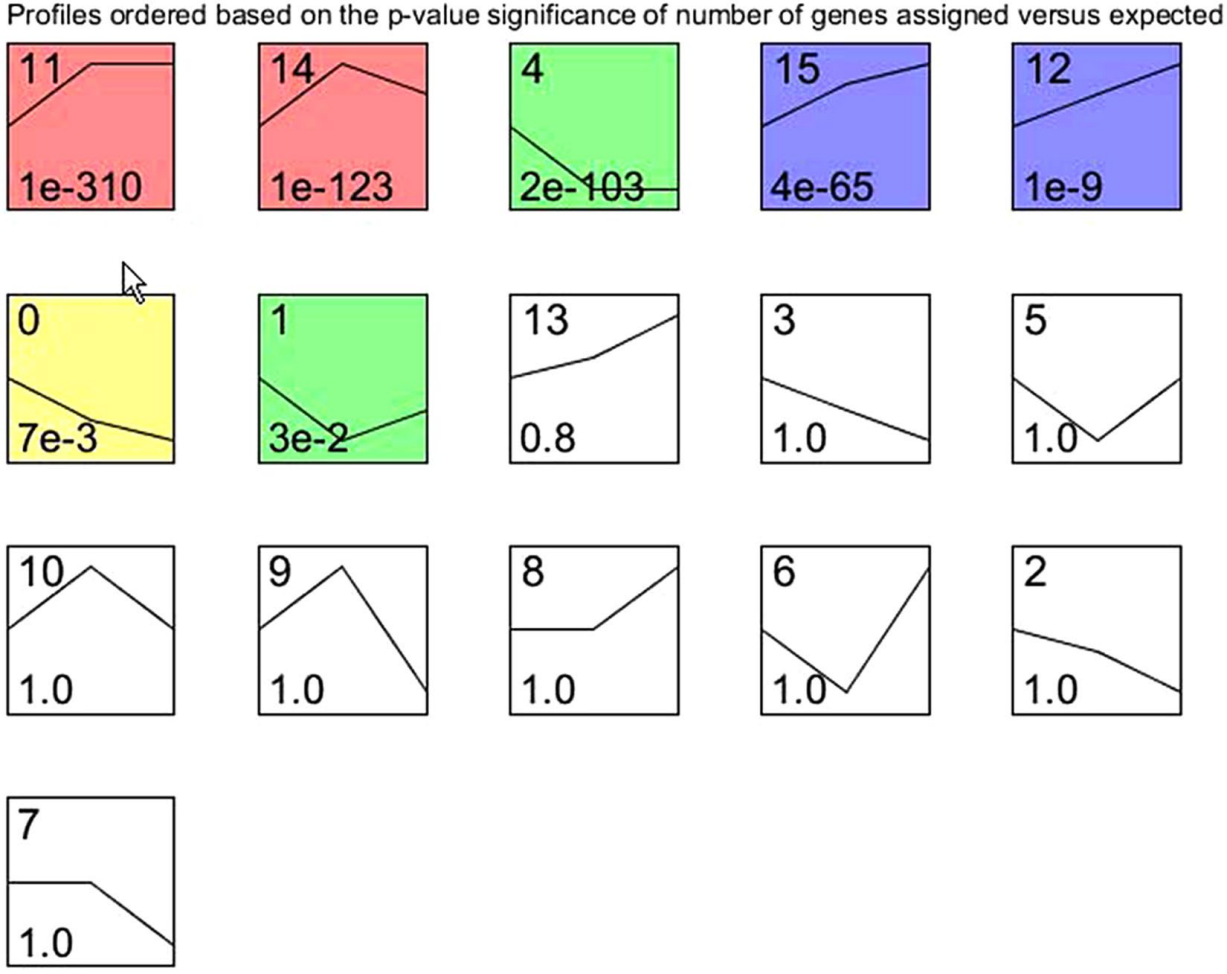
Maps of model profiles during development of tongue cancer in C57BL/6 J mice. The model profiles derived from STEM are ordered by the P value during lingual carcinogenesis in C57BL/6 J mice. Each box corresponds to a model expression profile. The number on the top of box is the profile ID. The line delineates the expression change of model profile at different time points, that is, C (0 week, the leftmost point), M (12 week, the middle point), and E (28week, the rightmost point), during experiment. The p-value is provided on the bottom of box. The colored profiles (i.e. profile 11, 14, 4, 15, 12, 0, and 1) present assignments with statistically significant number of genes.

Seven standard profiles showed statistical significance with algorithm of STEM (Fig. 2). Among these, profile 11 had the highest statistical significance (P = 1.1E-340). There were 1 682 genes assigned to model profile 11. According to the expression patterns of genes assigned to this profile, both M vs. C and E vs. C subsets presented upregulation. In contrast, gene expression patterns remained unchanged between M and E. Hub genes and DEG subsets E vs. C upregulation (EvC up) and M vs. C upregulation (MvC up) matched profile 11. Thus, an intersection of 24 candidate genes was identified from these three gene sets. Accordingly, the six other profile intersections were also obtained.

All 63 hub genes extracted from the seven profile intersections with an MCC score ≥10 is shown in Table 1. Statistically significant GO terms involving candidate genes were obtained according to expression trends between C, M, and E (see Supplementary Table S2). In addition, KEGG pathways associated with candidate genes were identified based on expression differences between C, M, and E (see Supplementary Table S3). The top five pathways involving the most numbers of candidate genes are listed in Table 2. The leading KEGG pathway was defined as “pathways in cancer” (see Supplementary Fig. S4).

**Table 1:** Hub Genes identified during lingual carcinogenesis.

	Gene symbol^a^	Gene ID^b^	MCC score^c^	GO	Pathway	P value of DRMP	Hub gene set
**Profile 11**							
1	*HDAC2*	HGNC:4853	62,92	426	7	å 0.05	EvCup, MvCup
2	*EP300*	HGNC:3373	47,37	705	16	å 0.05	EvCup, MvCup
3	*RAC1*	HGNC:9801	35,35	514	19	å 0.05	EvCup, MvCup
4	*CRK*	HGNC:2362	34,38	196	11	å 0.05	EvCup, MvCup
5	*TBP*	HGNC:11588	28,28	186	4	0.046	EvCup, MvCup
6	*SMAD1*	HGNC:6767	24,28	342	3	0.009	EvCup, MvCup
7	*ABI1*	HGNC:11320	23	74	0	å 0.05	EvCup
8	*SMAD4*	HGNC:6770	23	257	8	0.0001	EvCup
9	*TGFBR1*	HGNC:11772	22,22	578	12	å 0.05	EvCup, MvCup
10	*CEBPA*	HGNC:1833	21,21	315	3	å 0.05	EvCup, MvCup
11	*CDC42*	HGNC:1736	21	325	16	å 0.05	MvCup
12	*H3F3A*	HGNC:4764	18,18	42	3	å 0.05	EvCup, MvCup
13	*SMARCB1*	HGNC:11103	17,17	277	0	å 0.05	EvCup, MvCup
14	*TNFRSF1A*	HGNC:11916	16	212	2	å 0.05	EvCup
15	*PDPK1*	HGNC:8816	16,16	499	12	0.024	EvCup, MvCup
16	*MAP2K1*	HGNC:6840	14	246	10	å 0.05	EvC up
17	*PFN1*	HGNC:8881	13	144	2	å 0.05	EvC up
18	*NFKB1*	HGNC:7794	12,11	281	21	å 0.05	EvCup, MvCup
19	*CASP7*	HGNC:1508	12	0	0	å 0.05	EvCup
20	*PLA2G4A*	HGNC:9035	11	161	0	å 0.05	EvCup
21	*SH3GL2*	HGNC:10831	10,10	80	1	å 0.05	EvCup, MvCup
22	*ATF4*	HGNC:786	10	99	4	å 0.05	EvCup
23	*APH1B*	HGNC:24080	10,10	70	0	å 0.05	EvCup, MvCup
24	*PKNOX1*	HGNC:9022	10	0	0	å 0.05	EvCup
**Profile 14**							
1	*STK38*	HGNC:17847	50,49 50	281	0	å 0.05	EvCup, MvCup, EvMdown
2	*CDK2*	HGNC:1771	41,58	137	8	å 0.05	EvCup, EvMdown
3	*KIT*	HGNC:6342	32	262	6	å 0.05	EvMdown
4	*LBR*	HGNC:6518	29,29 29	102	0	å 0.05	EvCup, MvCup, EvMdown
5	*DAB1*	HGNC:2661	26	174	0	å 0.05	EvM down
6	*CAMK2A*	HGNC:1460	22,26, 21	327	18	0.0051	EvCup, MvCup, EvMdown
7	*ACTG1*	HGNC:144	17,17	114	11	å 0.05	EvCup, MvCup
8	*HDAC4*	HGNC:14063	17	231	2	å 0.05	EvMdown
9	*ITGB1*	HGNC:6153	16,16	497	9	å 0.05	EvCup, MvCup
10	*NR3C1*	HGNC:7978	15,17	413	0	å 0.05	EvCup, MvCup
11	*LEPR*	HGNC:6554	15	73	1	å 0.05	EvMdown
12	*CCND2*	HGNC:1583	14	88	6	å 0.05	EvMdown
13	*MTA1*	HGNC:7410	14,15	71	0	å 0.05	EvCup, EvMdown
14	*SALL4*	HGNC15924	14,60	131	0	å 0.05	EvCup, EvMdown
15	*PFN2*	HGNC:8882	13	0	0	å 0.05	EvCup
16	*ERBB2*	HGNC:3430	12	40	0	å 0.05	MvCup
17	*ATXN3*	HGNC:7106	11,11	191	1	0.0273	EvCup, MvCup
18	*NDEL1*	HGNC:17620	11	199	0	å 0.05	EvMdown
19	*CAV1*	HGNC:1527	11	385	4	å 0.05	EvMdown
20	*CBLB*	HGNC:1542	10	88	5	å 0.05	EvMdown
**Profile 4**							
1	*CYLD*	HGNC:2584	26,50	268	0	å 0.05	EvCdown, MvCdown
2	*GRIN2B*	HGNC:4586	23	108	0	å 0.05	MvCdown
3	*DAXX*	HGNC:2681	14	57	0	å 0.05	MvCdown
4	*CDH2*	HGNC:1759	11	106	0	0.0011	MvCdown
**Profile 15**							
1	*EWSR1*	HGNC:3508	49,49	141	1	å 0.05	MvCup, EvMup
2	*SMC3*	HGNC:2468	26	111	2	å 0.05	EvCup
3	*HIST1H4C*	HGNC:4787	14	0	2	å 0.05	EvCup
4	*LIN7C*	HGNC:17789	12	26	0	å 0.05	EvCup
5	*TET2*	HGNC:25941	10	30	0	å 0.05	MvCup
**Profile 12**							
1	*PPP1CC*	HGNC:9283	52,52	173	8	å 0.05	EvCup MvCup
2	*PCNA*	HGNC:8729	23	122	4	å 0.05	EvCup
3	*ELOC*	HGNC:11617	16	81	4	å 0.05	EvCup
**Profile 0**							
1	*RYR1*	HGNC:10483	19	91	0	å 0.05	EvCdown
2	*NOTCH1*	HGNC:7881	15,15, 15	598	2	å 0.05	EvCdown, MvCdown, EvMdown
**Profile 1**							
1	*PRNP*	HGNC:9449	47,47	317	1	å 0.05	MvCdown EvMup
2	*PPP2CA*	HGNC:9299	19,20	190	4	å 0.05	MvCdown EvMup
3	*APC*	HGNC:583	15	182	3	å 0.05	MvCdown
4	*HDAC6*	HGNC:14064	14,14	286	0	å 0.05	EvCdown, MvCdown
5	*USP8*	HGNC:12631	12	51	0	å 0.05	EvMup
total	63						

63 candidate genes are identified as an intersection out of analyses of STEM, Cytoscape plugin cytoHubba and differentially expressed genes screening. Candidate genes of significant profiles are listed according to their MCC scores. GO is the number of Gene Ontology terms in which candidate genes are involved. Pathway represents the number of KEGG pathways with which candidate genes are associated.

Hub gene set: the subset of Hub gene from which candidate gene is selected. EvCup = E vs. C upregulation, EvCdown = E vs. C downregulation.

^a^Orthologous gene of Homo sapiens annotated in HUGO Gene Nomenclature Committee(HGNC), ^b^gene ID of HGNC, ^c^If a gene could be identified in more than one Hub gene set, MCC score is listed in line with the order in the column of Hubgene set, DEG: differentially expressed gene, DRMP: differentially regulated methylation of promoters.

**Table 2:** Top five pathways associated with candidate genes(CGs).

	PathwayID^a^	Definition	Number^b^	Symbol of CGs^c^
1	mmu05200	Pathways in cancer	13	*HDAC2*, *EP300*, *RAC1*, *CRK*, *TGFBR1*, *CEBPA*, *NFKB1*, *SMAD4*, *CDC42*, *ITGB1*, *ELOC*, *APC*, *MAP2K1*
2	mmu05203	Viral carcinogenesis	9	*HDAC2*, *EP300*, *RAC1*, *TBP*, *NFKB1*, *CDC42*, *CDK2*, *HDAC4*, *CCND2*
3	mmu04810	Regulation of actin cytoskeleton	9	*RAC1*,*CRK*,*PFN1*,*CDC42*,*ACTG1*,*ITGB1*,*PPP1CC*,*APC*, *MAP2K1*
4	mmu04510	Focal adhesion	9	*RAC1*, *CRK*, *PDPK1*, *CDC42*, *ACTG1*, *ITGB1*, *CCND2*, *CAV1*, *PPP1CC*
5	mmu05205	Proteoglycans in cancer	8	*RAC1*, *PDPK1*, *CDC42*, *CAMK2A*, *ACTG1*, *ITGB1*, *PPP1CC*, *MAP2K1*

The top five pathways are listed according to the number of candidate genes involved in (www.kegg.jp/kegg/kegg1.html)^30,31^.

^a^PathwayID: Pathway identifiers used in KEGG, ^b^number of candidate genes involved in corresponding pathway, ^c^orthologous gene of Homo sapiens annotated in Entrez Gene.

Thirty-five and 19 candidate genes were differentially expressed in subsets of M vs. C and E vs. M. Compared with the total DEGs identified in the respective gene subsets, statistical differences existed between hub genes expressed in the M vs. C and E vs. M subsets (chi-square test, α = 0.05, χ^2^ = 6.08, P < 0.025).

### Validation of microarray data by quantitative reverse transcription-PCR (qRT-PCR)

**Figure 3:**
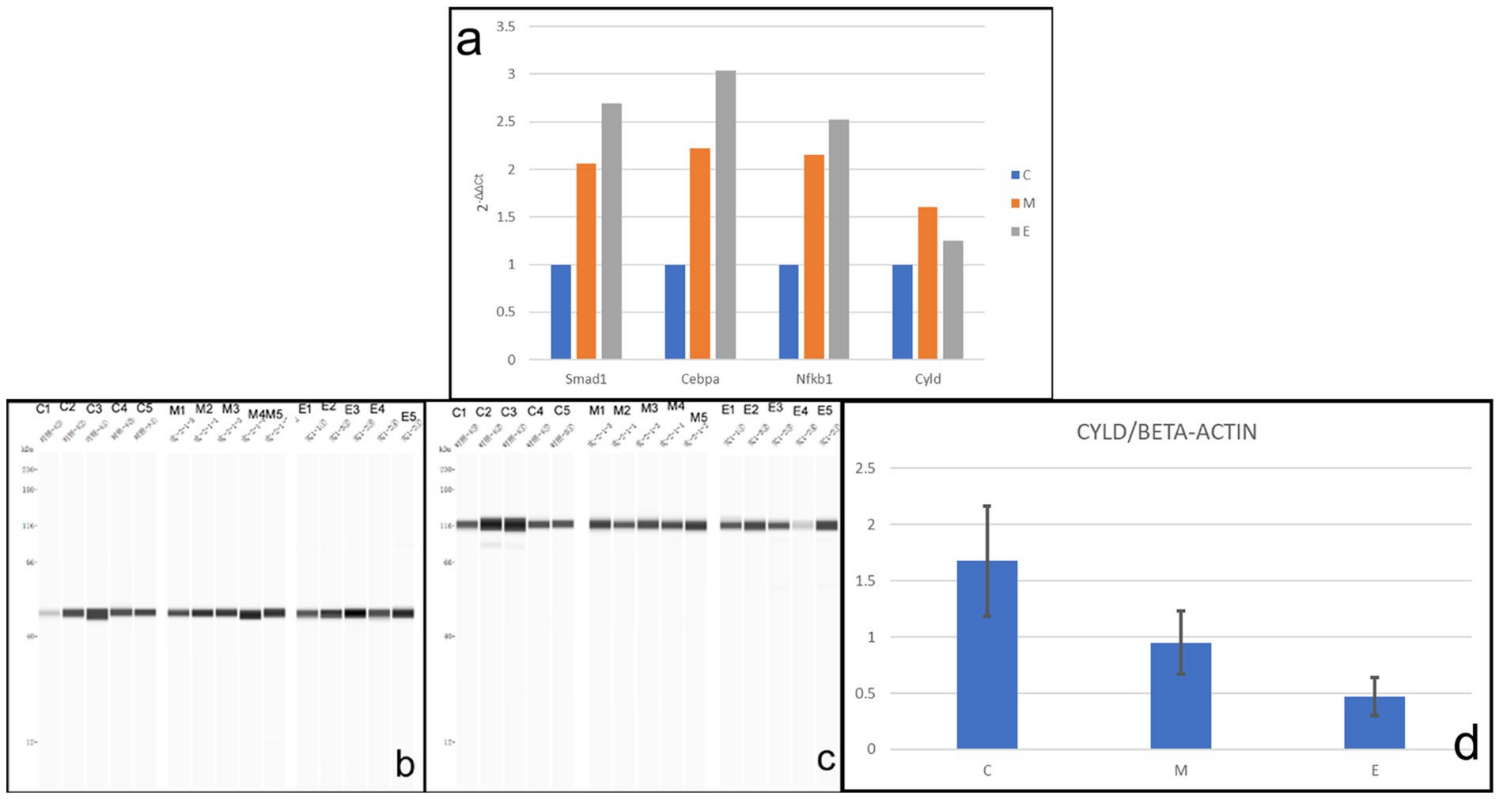
Results of qRT-PCR and WES. **(a)** The expressions of mRNAs of *Smad1*, *Cebpa*, *Nfkb1* and *Cyld* are normalized to *Gadph* and calculated using the 2^−ΔΔCt^ method. The mRNA alterations of *Smad1*, *Cebpa*, and *Nfkb1* by qRT-PCR are identical to those determined by microarray. But the mRNA expression of *Cyld* by qRT-PCR presents upregulation and is opposite to those determined by microarray. (**b**–**d**) Chemiluminescent images of capillary with beta actin **(b)** and Cyld **(c)** had been assayed by Compass software individually and given as a lane vie (The images of C, M, and E lanes which were obtained in different experiments now are grouped and delineated with white space in **b** and **c**. The original images were supplied in supplementary information). The expression of Cyld was normalized by beta actin and was calculated at the value of Cyld divided by that of beta actin in each sample. The results of WES were evaluated using analysis of variance (**d**, N = 5; error bars represent ± standard deviation). The result demonstrates that Cyld is statistically significantly down-regulated between C, M, and E (ANOVA, α = 0.05, F = 15.58, P < 0.01). C1–C5: samples from animals of 0th week. M1–M5: samples from animals of 12th week. E1–E5: samples from animals of 28th week.

The mRNA levels of *Smad1*, *Cebpa*, *Nfkb1*, and *Cyld* were verified by qRT-PCR. Expression variations in *Smad1*, *Cebpa*, and *Nfkb1* were identical to those in the microarray. However, *Cyld* expression change by qRT-PCR showed an upregulatory trend (Fig. 3). Thus, the expression of *Cyld* was further assayed with WES, which demonstrated that, compared with expression in C, *Cyld* was significantly downregulated in M and E (see Supplementary Table S4, Fig. 3).

Notably, 35/63 (55.5%) and 27/63 (44.5%) candidate genes were abnormally expressed in human oral cancer and other cancer types, respectively^6,10–22^.

### Gene promoter methylation analysis

Methylated DNA immunoprecipitation sequencing (MeDIP-Seq) was carried out to determine potential changes in the methylation patterns of gene promoters in our SCC mouse model. A total of 3,889 gene promoters with significant differential methylation were identified (see Supplementary Table S5). Significantly altered promoter methylations were identified in seven hub genes (*Tbp*, *Smad1*, *Smad4*, *Pdpk1*, *Camk2*, *Atxn3*, and *Cdh2*) (Table 1). MeDIP-Seq raw data have been deposited into GEO (GSE102488).

### Immunohistochemistry and correlation analyses in clinical SCC specimens

**Figure 4:**
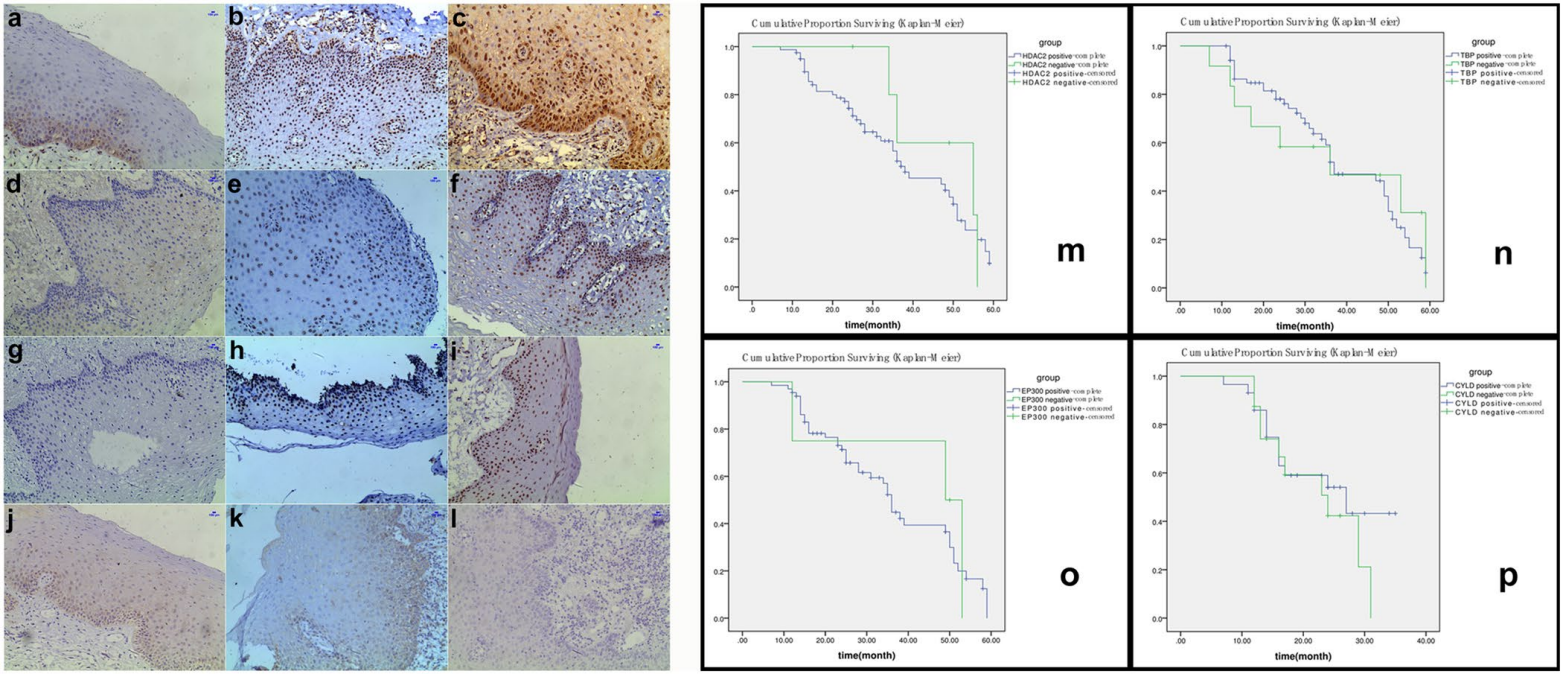
Immunohistochemical results and Kaplan–Meier survival curves of HTSCCs patients. (**a**–**l**) The expressions of HDAC2, TBP, EP300 and CYLD in samples of HTSCCs, paired normal mucosa, and OPL were assayed by immunohistochemical staining and demonstrated with a magnification of ×100. HDAC2, TBP and EP300 present nuclear staining. CYLD demonstrates nuclear, cytoplasmic and plasmalemmal staining. The length of blue bar in the top right corner of histological sections is 100 μm. (**a**,**d**,**g**,**j**) paired normal mucosa; (**b**,**e**,**h**,**k**) OPL; (**c**,**f**,**i**,**l**) HTSCCs. HDAC2: (**a**–**c**); TBP: (**d**–**f**); EP300: (**g**–**i**); CYLD: (**j**–**l**). (**m**–**p**) The cumulative survival curves of 85 patients with HTSCCs. The cumulative survival of patients with positive and negative expressions of HDAC2 (χ^2^ = 0.401, p = 0.527), TBP (χ^2^ = 0.014, p = 0.906), EP300 (χ^2^ = 0.145, p = 0.704,) and CYLD (χ^2^ = 0.750, p = 0.386) presented no significantly difference.

Analysis of clinical HTSCC and OPL specimens revealed 77, 68, 66, and 29 cases with positive immunohistochemical staining for HDAC2, TBP, EP300, and CYLD, respectively (Fig. 4). Except for CYLD, the levels of these proteins were significantly different between HTSCCs and normal mucosa. Significant differences were also found for TBP level between OPL and mucosa and for HDAC2 and EP300 level between HTSCCs and OPL. Notably, the levels of the preceding four proteins were unrelated to smoking history and lesion differentiation grade (see Supplementary Table S6). In addition, the overall survival of HTSCC patients was not correlated with HDAC2, TBP, EP300, and CYLD level (Fig. 4).

## Discussion

Transcript expression analysis of the 4NQO model of lingual tumorigenesis yielded 63 candidate genes that scored ≥10 by MCC and conformed to seven statistically significant profiles in the STEM algorithm. These genes potentially contribute to biological networks related to experimental tongue carcinogenesis and may also serve as diagnostic and/or therapeutic targets for tongue cancer.

The gradual, multi-stage histopathological process of oral tumorigenesis suggested that it was imperative to study the dynamics of gene expression in a stepwise manner. In the present study, differential gene expression patterns in tongue SCC were evaluated at baseline (C), early (M), and advanced (E) stages. Compared with the total number of DEGs, the number of differentially expressed hub genes was significantly greater in the early stage (M vs. C) than in the advanced stage (E vs. M). This suggested that important genetic alterations occurred at an early stage, although the corresponding pathological manifestations remained mild, compared with the gross lesions and severe symptoms characteristic of advanced stage. Thus, a more thorough understanding and further consideration of alterations in the early stage of tongue cancers are warranted for the development of early prevention strategies for patients at potential risk. Strikingly, 19/63 (30.16%) candidate genes could only be identified in M vs. C and E vs. M hub gene sets rather than in E vs. C. This indicated that when a comparison is made only between normal tissue and advanced tumors, approximately 30% of important genetic alterations are summarily omitted. The above results implied that a three-time-point assessment strategy for dynamic SCC-related gene expression is better than two-point comparisons, i.e., between normal tissues and advanced lesions.

Among the 63 candidate genes identified in our mouse model, 100% of the corresponding human orthologous genes were reported as relevant to human cancers, and up to 55.5% were associated with human oral cancer. These results indicated that the candidate genes identified from the mouse model used herein did not merely represent a subset of specific alterations in murine lingual SCCs but instead support this mouse model as a powerful tool to predict genetic alterations in human tongue cancer. 4NQO is usually considered a surrogate of typical chemical carcinogens such as tobacco. However, whether genetic discrepancies exist between smoking and non-smoking oral cancer patients remains controversial. In the present study, four orthologous candidate genes, *HDAC2*, *TBP*, *EP300*, and *CYLD*, demonstrated no significant expression differences between smoking and non-smoking HTSCC patients. Our results were consistent with prior research^23^ and indicated that the candidate genes identified do not—at least not entirely—represent a specific subset related to tobacco use. In comparison, Foy *et al*. suggested that the ES of genes differentially expressed between tumor and normal mucosa in the 4NQO CBA mouse model were higher in smokers and drinkers compared with that in never-smokers and never-drinkers and that the 4NQO model may be pertinent to smoking-associated HOSCCs^5^. Two possible reasons exist for the variance between the present results and those of Foy *et al*. Firstly, the experiments were aimed at different targets. In the present study, we focused on the comparison of hub genes instead of DEGs as a whole. Secondly, the different sampling sizes between studies may also underlie such different conclusions.

In this study, the pathways with significant ES for DEGs were used for further assessment of the involvement of candidate genes. We identified the top five pathways in which at least eight hub genes were involved. The leading pathway, associated with 13 hub genes, was “pathways in cancer,” which is a KEGG term with a complex network annotation composed of a series of signaling cascades including extracellular matrix–receptor interaction, focal adhesion, and apoptosis, among others. This was consistent with the results of two recent studies^5,6^. Similar results regarding the candidate genes involved in these pathways were reported in HOSCCs^10^. In another study, it was also reported that HDAC2 expression led to invasion/migration of human oral cancer cell lines via HIF-1α stability regulation^24^.

In turn, *Rac1* was found to be involved in tube formation to facilitate metastasis of HOSCCs via the adherens junction pathway^25^. Activation of *Rac1* was induced by either an epidermal growth factor receptor (EGFR)-based autocrine loop or as a consequence of oncogenic mutation of the *H-RAS* proto-oncogene. The EGFR/VAV2/RAC1 axis serves as a crucial pathway for the acquisition of motile and invasive properties in most head and neck SCC cells^26^. The Rho family of small GTP-binding proteins balances counteracting apoptotic and anti-apoptotic pathways through JNK and the transcriptional activation of NF-κB cascades, respectively, to promote cell survival or death^27^.

In our HTSCC specimens, *HDAC2* expression was not associated with tumor differentiation. This result was in line with a previous report^23^, but unlike that study, ours found no association between *HDAC2* expression and overall survival. This discrepancy is likely owing to differences in patients’ clinical stages at the time of surgery.

In summary, our three-time-point strategy was well suited to model and study characteristic histopathological and gene expression alterations seen in lingual tumorigenesis and appeared preferable to simple comparison of tumors and normal tissues to uncover the molecular pathogenesis of HOSCCs. By implementing both conventional and methylation array assays, combined with STEM, hub gene identification, and DEG criteria for ascertaining the dynamics of gene expression and epigenetic alterations during tongue carcinogenesis, we identified 63 candidate genes, which may serve as potential targets for preventive, diagnostic, and/or therapeutic approaches to HOSCCs.

## Material and Methods

### Tumor induction

This investigation was conducted in accordance with the Declaration of Helsinki, ARRIVE guidelines, and EU Directive 2010/63/EU for animal experiments and approved by the Medical Ethics Committee of Kunming Medical University. Female C57BL/6J mice were solely used for animal tests to prevent pregnancy interference and loss due to fighting among male mice. Specific pathogen-free (SPF), 4-week-old female C57BL/6J mice were purchased from the Medical Experimental Animal Center of Guangdong (Guangzhou, China). 4NQO was purchased from Sigma-Aldrich (St. Louis, MO, USA) and dissolved in propylene glycol. A total of 5, 5, and 75 mice were included in the distilled water control group, propylene glycol control group, and 4NQO experimental group, respectively. Water with 4NQO at a dose of 50 mg/L was administered to animals in the experimental group for 16 weeks. The drinking water was then exchanged for distilled water from week 17 through week 28. The tongues were excised and cut longitudinally in half, with one section used for immunohistochemistry and the other for microarray, MeDIP-Seq, qRT-PCR, and automated capillary western blot (WES).

### Histological examination

Serial sectioning at 4-μm thickness was performed longitudinally. The sections were stained with hematoxylin and eosin. For histological diagnoses, criteria were applied as previously described^28^.

### Microarray assay

We utilized nine samples for the microarray. Total RNA (1 μg) from the lingual mucosa of mice sacrificed at 0, 12, and 28 weeks was obtained using TRIzol (Invitrogen, Carlsbad, CA, USA). After amplified and labeled, Total RNA was hybridized onto a Whole Mouse Genome Oligo Microarray (4 × 44 K, v2, Agilent). The resulting text files were normalized with the GeneSpring GX v11.5.1 software package (Agilent). After quantile normalization, genes that had flags and detected in at least six out of nine samples were chosen for further analysis. Genes with a fold change (FC, log2 scaled) ≥2.0 and P ≤ 0.05 between two groups were identified as differentially expressed genes (DEGs). Functional analysis of DEGs was performed using gene ontology (GO) (http://www.geneontology.gov/)^29^ and the KEGG PATHWAY Database (http://www.genome.jp/kegg/pathway.html)^30,31^. GO analysis covers three domains: Biological Process, Cellular Component, and Molecular Function. GO and pathway analyses were performed according to gene expression trends between the 0^th^ week group (C), 12^th^ week group (M), and 28^th^ week group (E). The STEM software program (v1.3.8) was implemented for the analysis of microarray gene expression data^32^. Hub genes were identified using the Cytoscape plugin cytoHubba (http://hub.iis.sinica.edu.tw/cytohubba)^33^.

### MeDIP-Seq

Genomic DNA was extracted and purified using a Qiagen DNeasy Kit (Qiagen, Hilden, Germany) and sonicated to approximately 200–900 bp using a Bioruptor sonicator (Diagenode, Denville, NJ, USA). The fragmented sample was ligated to Illumina’s genomic adapters using a Genomic DNA Sample Kit (#FC-102-1002, Illumina, San Diego, CA, USA). Approximately 300–1,000-bp ligated DNA fragments were further immunoprecipitated with an anti-5-methylcytosine antibody (Diagenode). Sequencing was performed on Illumina HiSeq. 2000. To quantify the DNA methylation level of any specific region in the genome, a methylation score was defined as the number of extended reads per kb^34^. Regions with an FC ≥ 1.5 and P ≤ 0.05 between two groups were identified as differentially methylated regions.

### Integrative assay

The results of STEM, hub gene, and DEG screening were combined for identification of candidate early diagnosis biomarkers and therapeutic targets. Firstly, genes assigned to STEM-derived statistically significant model profiles were chosen for further analysis. In addition, the expression patterns of genes assigned to the profile (i.e., upregulation, downregulation, or steady-state) were visualized on the model profiles graph. Secondly, hubgs subsets were selected according to gene expression patterns in C, M, and E. Thirdly, hubgs were ordered according to MCC score. Those that scored ≥10 were selected for further assays. Fourthly, head-to-head comparisons were performed to reveal overlap between genes assigned to a specific STEM profile and their corresponding hub gene subset. Finally, the intersections were further filtered according to the DEG criteria mentioned above. The genes identified were thus considered important candidate molecules for early diagnosis and therapeutic targets for further study.

### qRT-PCR

Total RNA was extracted from 15 samples from C, M, and E. The first step of RT reaction involved addition of nuclease-free H_2_O to 0.5 μg RNA and 2 μL of 4 × gDNA Wiper Mix to a volume of 8 μL. Reactions were performed in a GeneAmp® PCR System 9700 (Applied Biosystems, Foster City, CA, USA) for 2 min at 42 °C. The second step involved addition of 2 μL of 5 × HiScript II Q RT SuperMix IIa. Reactions were performed for 10 min at 25 °C; 30 min at 50 °C; and 5 min at 85 °C. The 10-μL RT reaction mix was then diluted 10 × in nuclease-free water and held at −20 °C. Real-time PCR was performed using a LightCycler®480 II Real-time PCR Instrument (Roche, Basel, Switzerland) with 10 μL PCR reaction mixture that included 1 μL cDNA, 5 μL of 2 × QuantiFast® SYBR®Green PCR Master Mix (Qiagen), 0.2 μL forward primer, 0.2 μL reverse primer, and 3.6 μL nuclease-free water. Reactions were incubated in a 384-well optical plate (Roche) at 95 °C for 5 min, followed by 40 cycles of 95 °C for 10 s, and 60 °C for 30 s. Each sample was run in triplicate. At the end of the PCR cycles, melting curve analysis was performed to validate the specific generation of the expected PCR product. The primer sequences were obtained from the NCBI database (see Supplementary TableS 7). The expression levels of mRNAs were normalized to *Gadph* and calculated using the 2^−ΔΔCt^ method^35^.

### WES

Western blotting was performed using WES (ProteinSimple, San Jose, CA, USA). Briefly, 8 μL diluted protein lysate was mixed with 2 μL of 5× fluorescent master mix and heated at 95 °C for 5 min. The samples, blocking reagent, wash buffer, primary antibodies, secondary antibodies, and chemiluminescent substrate were dispensed into designated wells in a microplate. The plate was loaded into the instrument, and protein was drawn into individual capillaries on a 25-capillary cassette. Protein separation and chemiluminescence were performed automatically on individual capillaries. Data were analyzed using Compass software (ProteinSimple). Anti-CYLD rabbit mAb (8462 T, CST, Danvers, MA, USA) and beta*-*actin rabbit mAb (4970 S, CST) were used as primary antibody and loading control.

### Immunohistochemistry

Informed consent was obtained from each subject of this experiment. Eighty-five HTSCC and another 48 cases of OPL specimens were collected from patients at the Department of Oral Maxillofacial Surgery of the First Affiliated Hospital of Kunming Medical University from Jan 2013 to Jun 2017. Sections (4-μm thick) of paraffin-embedded tongue SCC tissues were dewaxed and rehydrated. Microwaving was used for antigen retrieval. Endogenous peroxidase activity was removed with 3% hydrogen peroxide for 10 min. Non-specific antibody binding was blocked with 10% sheep serum for 30 min. The sections were incubated for 1 h at 25 °C with the following primary antibodies: mouse monoclonal anti-HDAC2 IgG2b (ab12169, Abcam), mouse monoclonal anti-TATA binding protein IgG2a (ab51841, Abcam), mouse monoclonal anti-KAT3B/p300 IgG1 (ab54984, Abcam), and rabbit multiclonal anti-CYLD IgG (ab137524, Abcam) at a dilution of 1:400, 1:250, 1:200, and 1:100, respectively. Sections were then incubated sequentially with goat secondary antibodies against rabbit and mouse immunoglobulins (Dako REAL^TM^ EnVision^TM^ Detection System, Peroxidase/DAB+, Rabbit/Mouse, K5007, Agilent) for 40 min and 3,3′-diaminobenzidine tetrahydrochloride for 5 min. Sections were counterstained with Harris’ hematoxylin. Samples incubated with phosphate-buffered saline instead of primary antibody were used as negative controls.

Immunohistochemical results were quantitatively evaluated with ImageJ (v1.51j8, http://rsb.info.nih.gov/ij). Red intensity index (Ri) was calculated with an integration interval of red intensity scale from 200 to 255^36^. Ri values were measured in five independent visual fields in every sample. The mean of Ri values (MRV) from five different visual fields in negative control slides served as a reference for statistical analysis. Samples with high Ri values that were statistically different from control MRV were considered positive. Cumulative survival was calculated with SPSS software (v19.0.0, SPSS Inc., Chicago, IL, USA).

### Equipment and settings

An Olympus BX51 microscope (batch number:9E8056) equipped with Olympus UPlanFL objectives (Olympus Corporation, Tokyo, Japan) and a GD-300C camera (GAOTONG PACS, Guangzhou, China) linked to GD-PIMS v2.0 software (GAOTONG PACS) was used for acquisition of histopathological and immunohistochemical images.

### Statistical analysis

The rank-sum test (Kruskal–Wallis and Nemenyi method) was used for evaluation of histopathological results. A t-test was used for evaluation of DEGs and differential methylation of promoter regions. Significance testing was used for the value of GO terms. The EASE method, Fisher’s exact test, and hypergeometric test were used to estimate the enrichment P-value of the KEGG pathway. The results of WES and Ri were evaluated using analysis of variance. A chi-square test was used to assess immunohistochemical results for HDAC2, TBP, EP300, and CYLD and hub genes alterations in the early and advanced stage. Cumulative survival was calculated with the Kaplan–Meier product-limit method using the log-rank test.

## Electronic supplementary material

Supplementary information

Supplementary dataset

## Data Availability

The datasets generated during and/or analysed during the current study are available in the GEO repository, https://www.ncbi.nlm.nih.gov/geo/query/acc.cgi?acc=GSE101469; https://www.ncbi.nlm.nih.gov/geo/query/acc.cgi?acc=GSE102488.
