# Supplementary material for: Alterations of 63 hub genes during lingual carcinogenesis in C57BL/6J mice
Source: Sci Rep. 2018 Aug 22;8:12626. doi: 10.1038/s41598-018-31103-3 (PMC6105652; doi:10.1038/s41598-018-31103-3)
Supplement: Supplementary file 1 — Supplementary information [file 41598_2018_31103_MOESM1_ESM.pdf]

Title: **Alterations of 63 hub genes during lingual carcinogenesis in C57BL/6J mice**

Hua Liu[a], Jianjiao Li[b], Ying Yang[c], Liu Liu[b], Lifu Yu[b], Minsong Tu[b], Ruihong Yuan[b], Wanyuan Yue[a], Qi Luo[a], Yonghua Ruan[d], Xiaoming Dai[b]

[a], Department of Oral and Maxillofacial surgery, the Fourth Affiliated Hospital of Kunming Medical University

[b], Maxillofacial Service of the Department of Plastic Surgery, the First Affiliated Hospital of Kunming Medical University,

[c], Department of Pathology, the First Affiliated Hospital of Kunming Medical University,

[d], Department of Pathology, Kunming Medical University

Supplementary Fig. S1 Top ten enrichment Score value of the significant enrichment terms of gene ontology

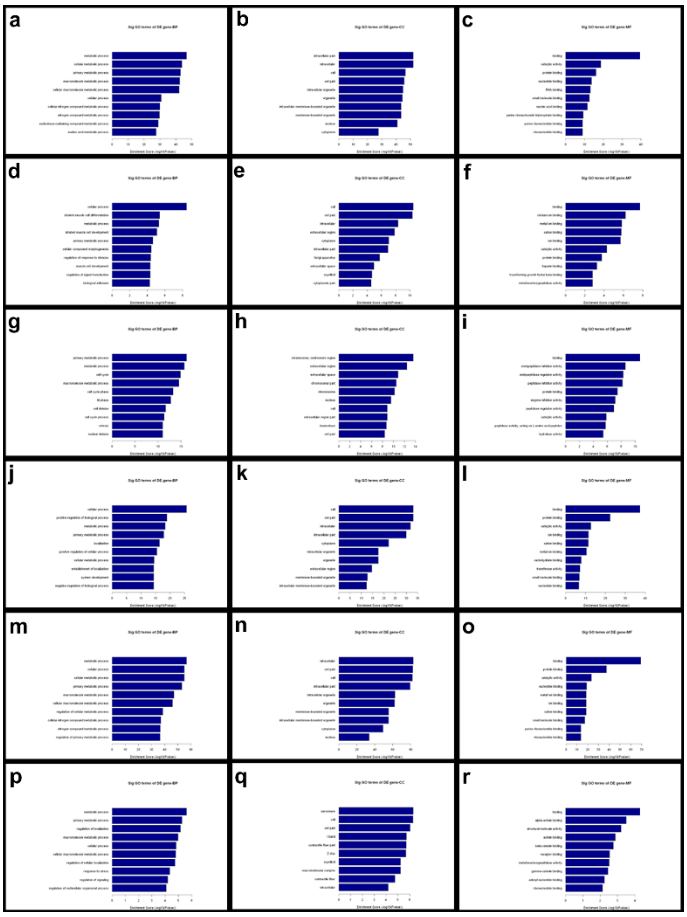

The top ten Enrichment Score value of the significant enrichment terms of gene ontology are delineated in this figure. Fisher’s exact test is used to find if there is more overlap between the DE list and the GO annotation list than would be expected by chance. The p-value denotes the significance of GO terms enrichment in the DE genes. The lower the p-value, the more significant the GO Term (p-value<=0.05 is recommended).

Enrichment Score: The GOID ‘s Enrichment Score value, it equals (-log10(Pvalue))

a-c: The top GO terms of Biological Process (a), Cellular Component (b), and Molecular Function (c) as to subset of EvCup (genes in E upregulated compared to C).

d-f: The top GO terms of Biological Process (d), Cellular Component (e), and

Molecular Function (f) as to subset of EvCdown (genes in E downregulated compared to C).

g-i: The top GO terms of Biological Process (g), Cellular Component (h), and Molecular Function (i) as to subset of EvMup (genes in E upwnregulated compared to M).

j-l: The top GO terms of Biological Process (j), Cellular Component (k), and Molecular Function (l) as to subset of EvMdown (genes in E downwnregulated compared to M).

m-o: The top GO terms of Biological Process (m), Cellular Component (n), and Molecular Function (o) as to subset of MvCup (genes in M upwnregulated compared to C).

p-r: The top GO terms of Biological Process (p), Cellular Component (q), and Molecular Function (r) as to subset of MvCdown (genes in M downwnregulated compared to C).

Supplementary Fig. S2 Top ten enrichment score value of the significant enrichment pathways

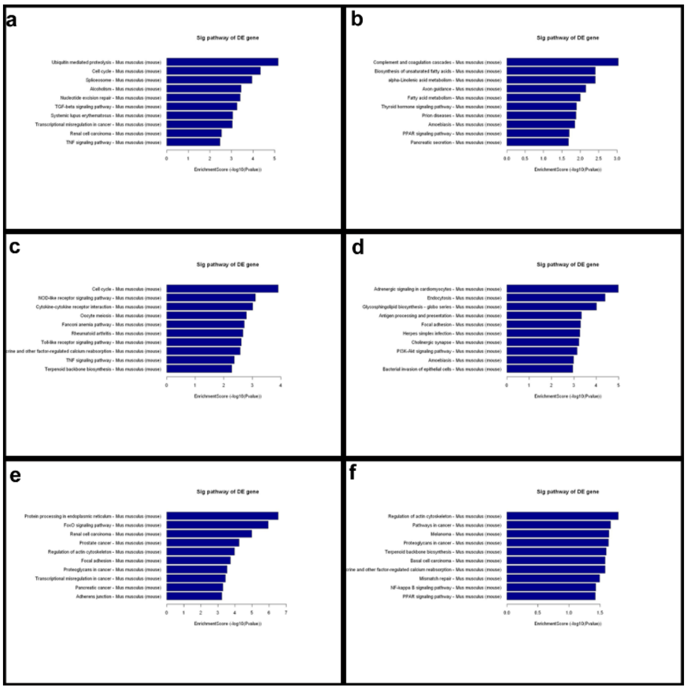

The bar plot shows the top ten Enrichment score value of the significant enrichment pathways in accordance to the genetic alterations between C, M and E. The Enrichment Score value of the PathwayID equals "-log10(P-value)”

a: The top ten significant enrichment pathways out of subset of EvCup (genes in E upregulated compared to C)

b: The top ten significant enrichment pathways out of subset of EvCdown (genes in E downregulated compared to C)

c: The top ten significant enrichment pathways out of subset of EvMup (genes in E upregulated compared to M)

d: The top ten significant enrichment pathways out of subset of EvMdown (genes in E downregulated compared to M)

e: The top ten significant enrichment pathways out of subset of MvCup (genes in M upregulated compared to C)

f: The top ten significant enrichment pathways out of subset of MvCdown (genes

in M downregulated compared to C)

Supplementary Fig. S3 Hub gene networks

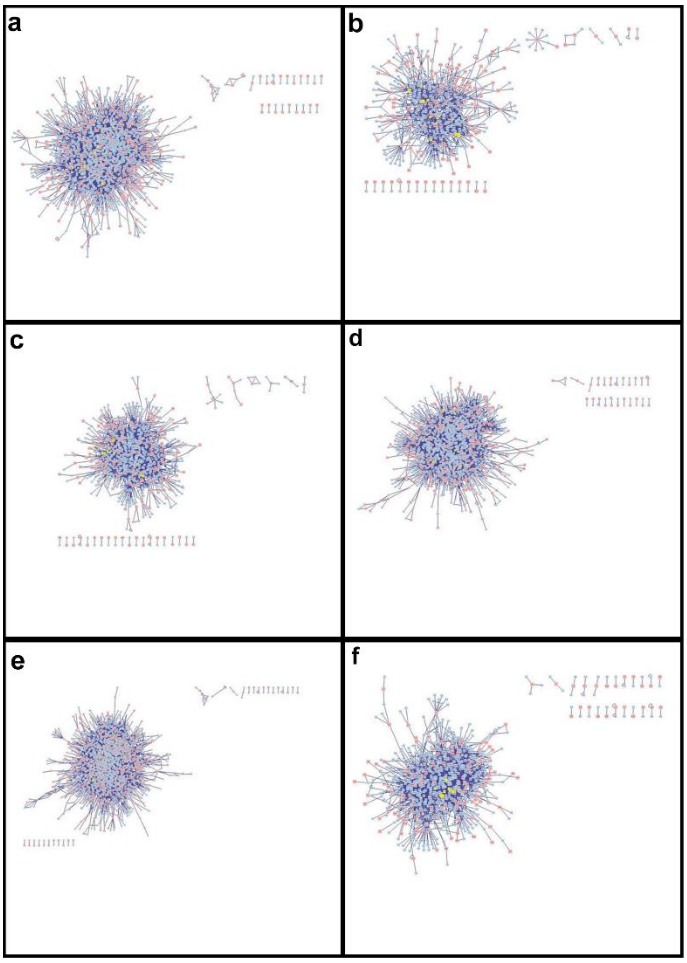

The sketch of hub gene networks is delineated with Cytoscape plugin cytoHubba in line with the trend of genetic expressions between C, M and E. Yellow nodes in the network are top 10 hub genes.

a: Hub gene network associated with subset EvCup ((genes in E upregulated compared to C).

b: Hub gene network associated with subset EvCdown ((genes in E downregulated compared to C)

c: Hub gene network associated with subset EvMup ((genes in E upregulated

compared to M).

d: Hub gene network associated with subset EvMdown ((genes in E downregulated compared to M)

e: Hub gene network associated with subset MvCup ((genes in M upregulated compared to C).

f: Hub gene network associated with subset MvCdown ((genes in M downregulated compared to C).

Supplementary Fig. S4 Top five statistically significant pathways map with enrichment for candidate genes

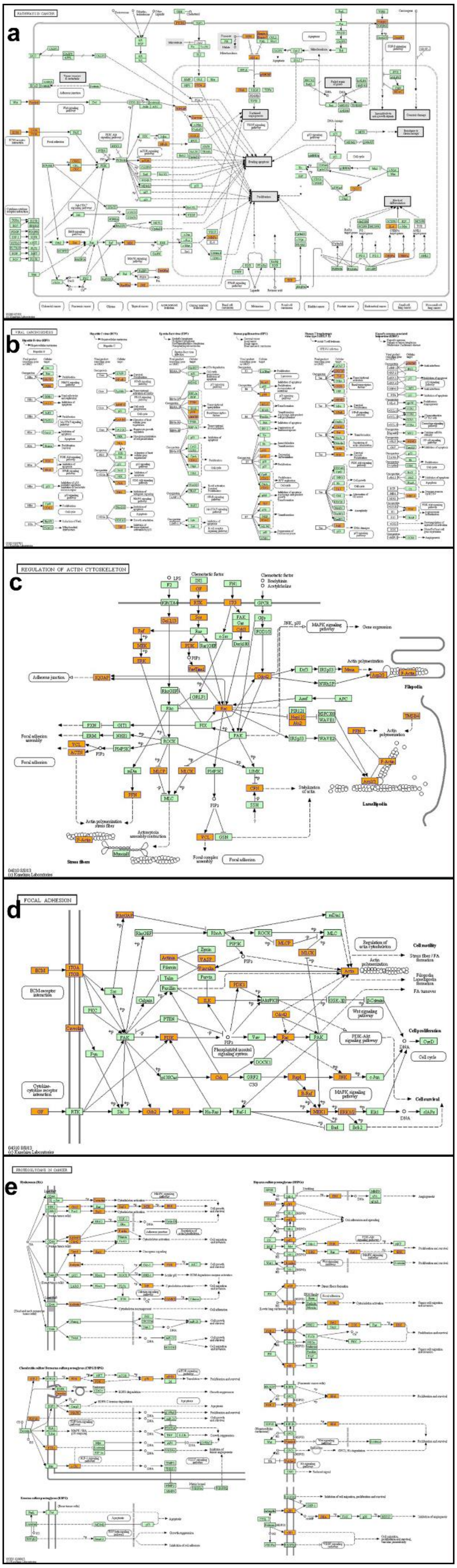
a
PATHWAYS IN CANCER
b
VIRAL CARCINOGENESIS
c
REGULATION OF ACTIN CYTOSKELETON
d
FOCAL ADHESION
e
PROTEOGLYCANS IN CANCER

The top five pathways involving the most numbers of candidate genes were demonstrated with KEGG imagery (www.kegg.jp/kegg/kegg1.html)[30,31]. Yellow marked nodes were associated with down-regulated genes. Orange marked nodes were associated with up-regulated or only whole dataset genes. Green nodes had no significance.

a: the pathway mmu05200 was defined as pathways in cancer - Mus musculus (mouse). b: the pathway mmu05203 was defined as viral carcinogenesis - Mus musculus (mouse).c: the pathway mmu04810 was defined as regulation of actin cytoskeleton - Mus musculus (mouse). d: the pathway mmu04510 was defined as focal adhesion - Mus musculus (mouse). e: the pathway mmu05205 was defined as proteoglycans in cancer - Mus musculus (mouse).

Supplementary Table S4 CYLD protein level between C, M and E

sample	CYLD/BETA-ACTIN(mean±standard deviation)	P value
C	1.68±0.49	<0.01
M	0.95±0.28	
E	0.46±0.17	

CYLD protein expressions were assayed by WES. BETA-ACTIN was used as loading control. The down-regulation of protein expressions of CYLD presents statistical significance between C, M, and E (ANOVA, $\alpha = 0.05$, $F = 15.58$, $P<0.01$).

Supplementary Table S6 Immunohistochemical staining of HDAC2, TBP, EP300, and CYLD

HDAC2						
	positive	negative	P value-a			
HTSCCs	77	8	**χ2=13.39**	**χ2=8.80**		**χ2=7.95**
OPL	34	14		**P<0.005**	**χ2=0.61**	
lingual mucosa	6	5	**P<0.005**		**P>0.25**	**P<0.005**
Smokers-b	41	2	χ2=2.59			
non-smokers-b	36	6	P>0.1			
Well-differentaition	52	5	χ2=0.11			
Moderately-and poorly-differentiation	25	3	P>0.9			

TBP						
	positive	negative	P value-a			
HTSCCs	68	12	**χ2=13.4**	**χ2=3.43**		**χ2=13.02**
OPL	31	13		**P>0.05**	**χ2=4.12**	
lingual mucosa	3	7	**P<0.005**		**P<0.05**	**P<0.005**
Smokers-b	35	5	χ2=0.4			
non-smokers-b	33	7	P>0.5			
Well-differentaition	46	8	χ2=0.04			
Moderately-and poorly-differentiation	22	4	P>0.9			

EP300						
	positive	negative	P value-a			
HTSCCs	66	4	χ2=26.2	χ2=18.6		χ2=32.3
OPL	22	14	P<0.005	P<0.005	χ2=0.74	
lingual mucosa	4	6			P>0.25	P<0.005
Smokers-b	35	1	χ2=1.28			
non-smokers-b	31	3	P>0.1			
Well-differentaition	44	2	χ2=0.02			
Moderately-and poorly-differentiation	22	2	P>0.9			

CYLD						
	positive	negative	P value-a			
HTSCCs	29	16	χ2=4.46	χ2=2.77		χ2=2.7
OPL	18	18	P>0.1	P>0. 05	χ2=0.41	
lingual mucosa	3	7			P>0. 5	P>0.1
Smokers-b	18	12	χ2=0.88			
non-smokers-b	11	4	P>0.25			
Well-differentaition	20	11	χ2=0.01			
Moderately-and poorly-differentiation	9	5	P>0.9			

IHC were performed to assay the levels of HDAC2, TBP, EP300 and CYLD between HTSCCs, paired lingual mucosa, and OPL. The expressions of

preceding proteins were also performed between smokers and nonsmokers among cases of HTSCCs as well as well-differentiated and moderately-and poorly-differentiated HTSCCs. $\chi2$ and p value were calculated as $\alpha = 0.05$.

a: Chi-square test was used to assess the IHC results, b: smokers and nonsmokers only refer to patients of HTSCCs.

P value between HTSCCs, paired lingual mucosa and OPL

P value between HTSCCs and OPL

P value between lingual mucosa and OPL

P value between HTSCCs and paired lingual mucosa

Supplementary TableS7 The primer sequences of qRT-PCR

gene	forward	reverse
Smad1	5’-CAA CCA AGA GTT TGC TCA GCT A-3’,	5’-TTC ACG AAG CTC ATC CGA AT-3’
Cebpa	5’-TGA GTG AGG CTC TCA TTC TTT-3’	5’-AGA CCC ACT ACT ACA TAC ACC-3’
Nfkb1	5’-TGG TGT GGA GAC ATC CTT C-3’	5’-TTC CTG CCC ATA ACC GTG-3’
Cyld	5’-GTT CTT TCT CCA TCA CTT GCT-3’	5’-TGT ACC GCT TAT CCA ACT ACA T-3’
Gadph	5’-TCA TCC CAG AGC TGA ACG-3’	5’-TCA TAC TTG GCA GGT TTC TCC-3’

The primer sequences were synthesized by Generay Biotech (Shanghai, China). The expression levels of mRNAs were normalized to *Gadph.*
