# Supplementary material for: Alterations of 63 hub genes during lingual carcinogenesis in C57BL/6J mice
Source: Sci Rep. 2018 Aug 22;8:12626. doi: 10.1038/s41598-018-31103-3 (PMC6105652; doi:10.1038/s41598-018-31103-3)
Supplement: Supplementary file 2 — Supplementary dataset [file 41598_2018_31103_MOESM2_ESM.zip › Supplementary Table S2 Gene Ontology(GO) terms in which candidate genes are involved.docx]

**Legend of Supplementary Table S2 Gene Ontology(GO) terms in which candidate genes are involved**

GO terms with significant enrichment of differentially expressed genes, with which candidate genes are associated, are identified and listed in this table according to the expressing tendencies between C, M and E.
