# Supplementary material for: Alterations of 63 hub genes during lingual carcinogenesis in C57BL/6J mice
Source: Sci Rep. 2018 Aug 22;8:12626. doi: 10.1038/s41598-018-31103-3 (PMC6105652; doi:10.1038/s41598-018-31103-3)
Supplement: Supplementary file 2 — Supplementary dataset [file 41598_2018_31103_MOESM2_ESM.zip › Supplementary Table S3 Pathway in which candidate genes are involved.docx]

**Legend of Supplementary Table S3 Pathway in which candidate genes are involved**

Pathways with significant enrichment of differentially expressed genes, in which candidate genes are involved, are demonstrated in this table in line with the trend of alterations between C, M and E (www.kegg.jp/kegg/kegg1.html)^30,31^.
