# Supplementary material for: Alterations of 63 hub genes during lingual carcinogenesis in C57BL/6J mice
Source: Sci Rep. 2018 Aug 22;8:12626. doi: 10.1038/s41598-018-31103-3 (PMC6105652; doi:10.1038/s41598-018-31103-3)
Supplement: Supplementary file 2 — Supplementary dataset [file 41598_2018_31103_MOESM2_ESM.zip › Supplementary Table S5 Differentially methylated promoters of genes.docx]

**Legend of Supplementary Table S5 Differentially methylated promoters of genes during lingual carcinogenesis**

The genomic DNA methylation was performed with MeDIP-Seq. Sequencing was performed on an Illumina HiSeq 2000. The differentially methylated regions were identified by determining the FC and P values of the t-test. Regions with an FC of ≥1.5 and a P value of ≤0.05 between 2 groups were identified as differentially methylated regions. The differential methylations of DNA in the regions of promoters of genes between C, M and E are demonstrated according to the trend of genetic alterations.
