# Supplementary material for: Alterations of 63 hub genes during lingual carcinogenesis in C57BL/6J mice
Source: Sci Rep. 2018 Aug 22;8:12626. doi: 10.1038/s41598-018-31103-3 (PMC6105652; doi:10.1038/s41598-018-31103-3)
Supplement: Supplementary file 2 — Supplementary dataset [file 41598_2018_31103_MOESM2_ESM.zip › Supplementary Table S1 Differentially expressed genes during lingual carcicnogenesis.docx]

**Legend of Supplementary Table S1 Differentially expressed genes during lingual carcinogenesis**

Samples from the 0th week group (C), the 12th week group (M), and the 28th week group (E) were used for genome-scale microarray analysis by Genome Oligo Microarray (4x44K, v2, Agilent). The differentially expressed genes are identified in accordance to the threshhold of fold change (log2 scaled) of ≥ 2.0 and p value of ≤ 0.05 between C, M and E. For M vs. C, 2339, 2539 and 2482 genes are significantly altered in M vs. C, E vs. M and E vs. C individually.

EvCup: E versus C upregulation, EvCdown: E versus C downregulation, MvCup: M versus C upregulation, MvCdown: M versus C downregulation, EvMup: E versus M upregulation, EvMdown: E versus M downregulation.
